# Use of silica-based homogeneously distributed gold nickel nanohybrid as a stable nanocatalyst for the hydrogen production from the dimethylamine borane

**DOI:** 10.1038/s41598-020-64221-y

**Published:** 2020-04-29

**Authors:** Oznur Alptekin, Betul Sen, Aysun Savk, Umran Ercetin, Sibel Demiroglu Mustafov, Mehmet Ferdi Fellah, Fatih Sen

**Affiliations:** 1 Department of Mechanical Engineering, Faculty of Engineering, Dumlupınar University, Evliya Çelebi Campus, 43100 Kütahya, Turkey; 2 Sen Research Group, Department of Biochemistry, Faculty of Art and Science, Dumlupınar University, Evliya Çelebi Campus, 43100 Kütahya, Turkey; 3 Department of Chemical Engineering, Bursa Technical University, Mimar Sinan Campus, 16310 Bursa, Turkey

**Keywords:** Catalysis, Nanoparticles

## Abstract

In this study, the effects of silica-based gold-nickel (AuNi@SiO_2_) nanohybrid to the production of hydrogen from dimethylamine borane (DMAB) were investigated. AuNi@SiO_2_ nanohybrid constructs were prepared as nanocatalysts for the dimethylamine borane dehydrogenation. The prepared nanohybrid structures were exhibited high catalytic activity and a stable form. The resulting nanohybrid, AuNi@SiO_2_ as a nanocatalyst, was tested in the hydrogen evolution from DMAB at room temperature. The synthesized nanohybrids were characterized using some analytical techniques. According to the results of the characterization, it was observed that the catalyst was in nanoscale and the gold-nickel alloys showed a homogenous distribution on the SiO_2_ surface. After characterization, the turn over frequency (TOF) of nanohybrid prepared for the production of hydrogen from dimethylamine was calculated (546.9 h^−1^). Also, the prepared nanohybrid can be used non-observed a significant decrease in activity even after the fifth use, in the same reaction. In addition, the activation energy (E_a_) of the reaction of DMAB catalyzed AuNi@SiO_2_ nanohybrid was found to be 16.653 ± 1 kJmol^−1^ that facilitated the catalytic reaction. Furthermore, DFT-B3LYP calculations were used on the AuNi@SiO_2_ cluster to investigate catalyst activity. Computational results based on DFT obtained in the theoretical part of the study support the experimental data.

## Introduction

Today, the decreasing fossil fuels, the enhancing environmental problems and the dependence on energy caused a much tendency towards alternative energy sources. Therefore, hydrogen as a clean, highly efficient and non-toxic energy storage chemical has influenced scientists^1–3^. However, problems with the storage of hydrogen continue. Storage of hydrogen in gas and liquid phase is very difficult and costly. Therefore, the orientation towards hydrogen removal from some hydrogen storage materials has begun. Recently, especially Sodium Borohydride (NaBH_4_) (SBH) and Ammonia borane (AB) derivatives have been used economically and high efficiency^4–6^. Sodium Borohydride (SBH) (NaBH_4_) has a high hydrogen content of 10.8% and its usage is easy. Ammonia borane derivatives (AB) are a great source of energy because they have a 19.6% hydrogen content and have high stability^7,8^. In the presence of a proper catalyst, ammonia borane derivatives have a very high capacity to produce hydrogen. Numerous catalysts were utilized to produce hydrogen from ammonia boranes (ABs). The use of dimethylamine-borane, which is an AB derivative in the dehydrogenation of nanohybrid showing high catalytic activity, is highly advantageous in terms of yield^9–25^. The identification of products released after the hydrogen production reaction of dimethylamine borane (Eq. 1) compared to the other AB product is relatively simple. In hydrogen production from DMAB, as the derivatives of other ABs, catalysts are usually required for f-metal complexes. For this purpose, f-metal nanohybrids have been widely used and have been studied to be highly efficient^15,26–33^. For this purpose, high efficiency, stable, silica (SiO_2_) based AuNi nanohybrid (AuNi@SiO_2_) was synthesized for use in dehydrogenation of dimethylamine borane. For the dehydrogenation of the dimethylamine borane, the ultrasonic reduction method was employed to synthesize homogeneously dispersed AuNi nanohybrid with silica support. Then, the characterization of the synthesized nanohybrid was carried out employing some analytical methods like XPS, TEM, XRD, and HR-TEM analytical techniques. The prepared AuNi@SiO_2_ nanohybrid, using the ultrasonic reduction, tested at different amount of catalyst concentration for hydrogen evolution in the dehydrogenation of DMAB reaction. In view of the values found, the activation values, catalytic activity, turnover frequency and reusability of the nanohybrid used were investigated. It is noteworthy that the AuNi@SiO_2_ nanohybrid synthesized exhibited, according to the result of these investigations, a relatively high cycle frequency compared to other nanocatalysts in the literature.

1 $$2{({{\rm{CH}}}_{3})}_{2}{{\rm{NBH}}}_{3}\,\underrightarrow{{\rm{AuNi}}@{{\rm{SiO}}}_{2}}\,{[{({{\rm{CH}}}_{3})}_{2}{{\rm{NBH}}}_{2}]}_{2}+2{{\rm{H}}}_{2}$$

## Experimental

In our study, it was aimed to prepare SiO_2_ based AuNi nanohybrid using the ultrasonic reduction method. For this purpose, 0.25 mmol AuCl_3_ and NiCl_2_ were mixed in the ultrasonic sonicator and 2.5 mmol SiO_2_ was added as support. The resulting mixture was mixed using a magnetic stirrer to homogenize. Then, superhydride was added to the resulting solution, and then color change in solution was observed. This shows us that the particles (Au^3+^ and Ni^2+^) are reduced and nanohybrid formed. The Au^3+^ and Ni^2+^ cations have high stability by reducing to Au (0) and Ni (0). Dimethylamine borane was added to the nanocatalyst synthesized at room temperature (25 °C) and hydrogen output was examined during the reaction. XPS, TEM, XRD, and HR-TEM analytical techniques were used to investigate the characterization, structural morphology, particle size and content nanoparticles. In addition, stability, catalytic activity, activation parameters, efficiency and cycle frequency of nanohybrid, which are intended to make the reaction more active during dehydrogenation entered by DMAB, were evaluated.

### Computational method

The theoretical calculations which are employed for the structures in this study have been based on Density Functional Theory (DFT)^34^. The software of Gaussian 09^35^ has been used with the B3LYP-Hybrid method^36,37^. The basis set of LanL2DZ has been utilized in calculations for Au and Ni atoms. The basis set which is utilized for C, O and H atoms (the rest atoms in the structures) in a cluster is 6-31G (d, p) basis set.

The SiO_2_ cluster used for calculations has Si atoms and Oxygen atoms. The cluster was modeled as (002) surface. The SiO_2_ cluster structure was shown in the Supporting Information in Fig. S1. Dangling bonds of the O atoms have been saturated with H atoms to obtain a neutral cluster. To obtain the AuNi@SiO_2_ cluster representing the AuNi@SiO_2_ catalyst, Au and Ni atom were used. In this study, all atoms of the structures were relaxed in the course of all DFT calculations. The details of the theoretical strategy utilized in the theoretical part of this study have been stated in Supporting Information (Tables S1 and S2).

## Results and Discussion

**Figure 1:**
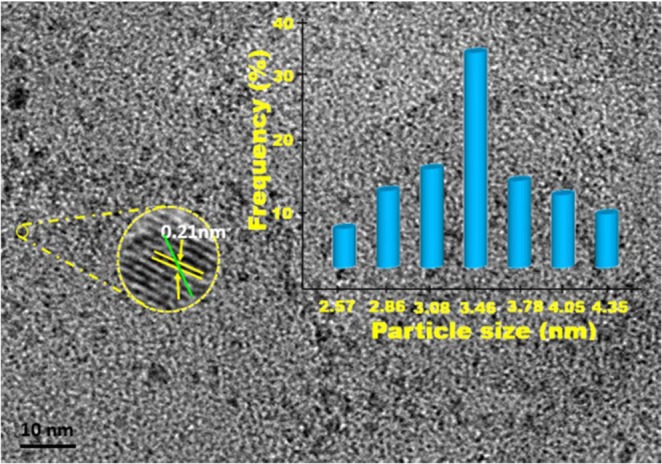
AuNi@SiO_2_ nanohybrid TEM, HR-TEM, and particle size histogram.

As a result of DMAB dehydrogenation reaction catalyzed with AuNi@SiO_2_ nanohybrid synthesized by ultrasonic method, structural analyzes of the nanocatalyst and its catalytic activity in hydrogen production were evaluated. XPS, TEM, XRD, B-NMR, SEM-EDX and HR-TEM techniques were used for the characterization of AuNi@SiO_2_ nanohybrid. Firstly, TEM and HRTEM methods were utilized to determine the morphology and particle size of nanohybrids. Figure 1 shows the synthesized nanohybrids as homogeneous and monodisperse on the SiO_2_ support material according to these methods. It was observed that the particles were not randomly clustered and accumulated and were stable in the spherical structure. According to these results, the particle size of AuNi@SiO_2_ nanohybrid was calculated to be 3.46 ± 0.89 nm. As shown in Fig. 1, HR-TEM was employed to show the nanohybrid AuNi@SiO_2_ synthesized atomic lattice fringes. When the results are compared with the literature, it has been confirmed that they are compatible with the literature. 11B-NMR study of the reaction showed that the dimethylamine borane (DMAB) and the catalytic dehydrogenation of dimethylamine borane in the presence of AuNi@SiO_2_ at room temperature (Supporting Information, Fig. S4).

**Figure 2:**
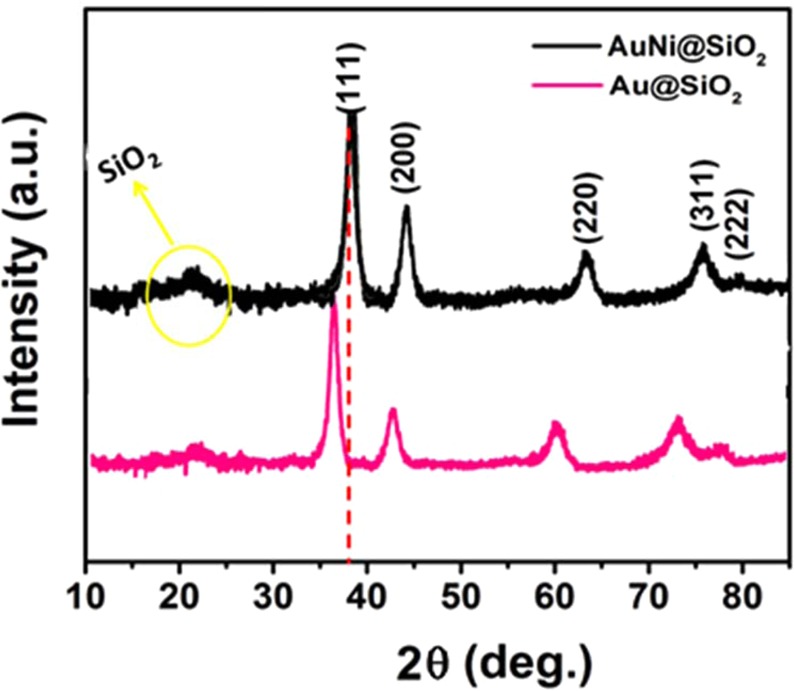
XRD patterns of Au@SiO_2_ and AuNi@SiO_2_ nanohybrid.

As a result of the introduction of Ni atoms into the Au lattice structure, the distortion of the lattice structure of gold occurs. Accordingly, the lattice parameters are expected to decrease after an alloy of AuNi formation. In order to explain this situation, the XRD curve of the nanohybrid having the highest active surface area was taken and compared with the XRD result of pure Au. The comparison of XRD curves is given in Fig. 2. Here, we see curves (111), (220) and (311) in the surface-centered cubic structure of gold. The most intense breaking peak for Au is (111). This value indicates that (111) is the most preferred growth direction of the Au core and then, is directly approved by HR-TEM. In Fig. 2, the 2θ = 38.3^o^ scatter curve for all nanohybrid is caused by the catalyst support material. In short, Fig. 2 demonstrates the formation of AuNi alloy nanohybrid materials. In Figs. S5 and S6, the XRD pattern and SEM image of after-use AuNi@SiO_2_ nanohybrids for H_2_ production is given in Supporting Information, respectively.

**Figure 3:**
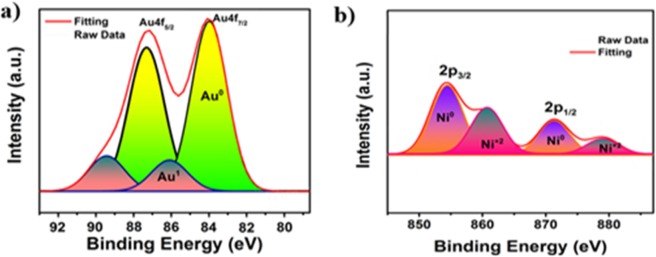
(**a**) XPS spectra of Au 4f and (**b**) Ni 2p AuNi@SiO_2_ nanohybrids.

For the characterization oxidation state of Au and Ni in the prepared catalyst sample, XPS analysis was performed. The determination of gold and nickel on the surface of the prepared nanohybrid structure is shown by the XPS results in Fig. 3; Ni-2p_3/2_ and Ni-2p_1/2_ peaks at 87.3 eV and 84.5 eV found in Au-4f_5/2_ and Au-4f_7/2_ peaks at 850.2 eV and 870.2 ev, respectively. Referring to Fig. 3, it can be said that most of the Au and Ni nanohybrid is zero-valued, and a small amount of Au^+3^ and Ni^+2^ valence. The lower energy shift of the gold and nickel binding XPS energies shows the character of the alloy. As a result of homogenous distribution of AuNi on SiO_2_ nanohybrid during synthesis, more zero oxidation peaks were observed. According to these data, we can explain that gold and nickel have a very good bonding structure and have a monodispers alloy nanohybrids. Furthermore, the survey spectra of AuNi@SiO_2_ nanohybrids were given in Supporting Information (Fig. S7). The results of XPS analysis for chemical composition and elemental percentages of AuNi@SiO2 nanohybrids were given in Supporting information (Tables S3 and S4). EDX analysis from four different areas and the element mapping results of the catalyst were given in Fig. S8 and Table S5 to better understand whether the synthesized catalyst forms alloy or core-shell.

**Figure 4:**
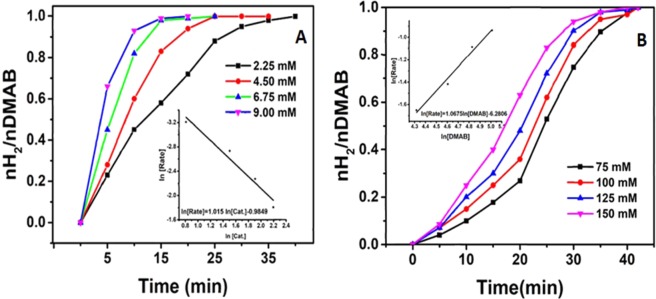
(**A**) DMAB graphical drawing of molar H_2_/mol against time in the existence of AuNi@SiO_2_ at varied catalyst concentrations at 25.0 ± 0.1 °C, (**B**) DMAB graphical drawing of molar H_2_/mol against time at different substrate concentrations catalyzed by AuNi@SiO_2_ for dehydrogenation of dimethylamine borane.

In our study, the efficacy of synthesized AuNi@SiO_2_ nanohybrid in the reactions of DMAB was tested. Figure 4A shows the amount of hydrogen produced by dehydrogenation of DMAB with different amounts of AuNi@SiO_2_ nanohybrid as 2.25, 4.50, 6.75 and 9.00 mM at 25.0 ± 0.1 °C. Figure 4A shows the hydrogen production rate and the linear part of each slope line for the catalytic reaction DMAB catalyzed AuNi@SiO_2_ nanohybrid. According to these calculations, the rate of hydrogen release is seen to grow as the catalyst amount increases. Also, in Fig. 4A, the slope of the plot is given as 1.015. According to this result, it is clear that the rate of reaction of hydrogen production depends on the amount of catalyst in the first order. Experiments to investigate the effect of dimethylamine borane on the catalytic reaction at room temperature were conducted using different amounts. The results obtained for this purpose are shown in Fig. 4B. According to these results, the amount of hydrogen produced by AuNi@SiO_2_ nanohybrid in different amounts (75, 100, 150 and 200 mM) was investigated. Also, in Fig. 4B, the slope of the plot is found as 1.0675. According to this result, it is clear that the rate of reaction of hydrogen production is dependent on the amount of DMAB in the first order.

**Figure 5:**
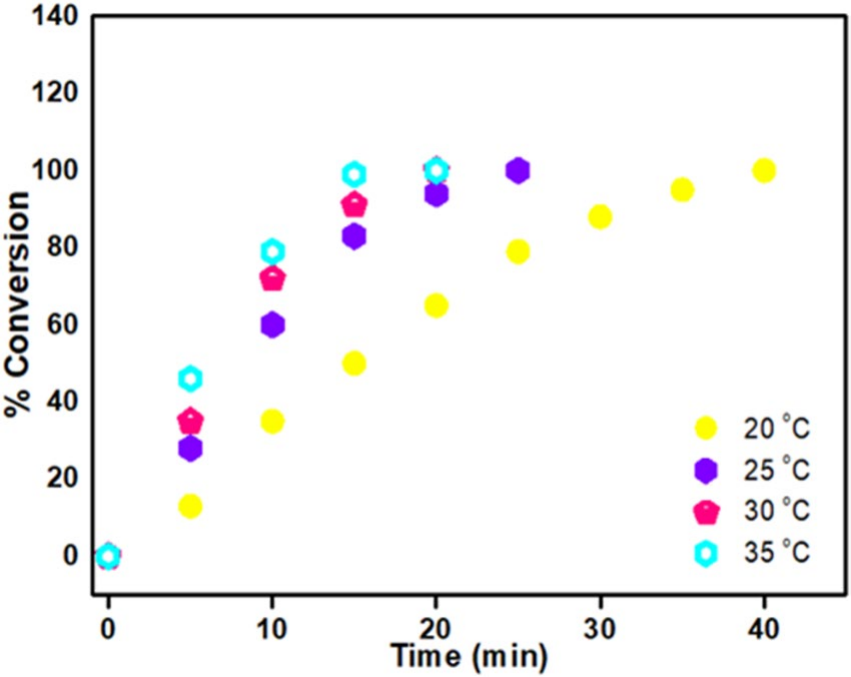
Time-dependent % conversion graph at different temperatures for dehydrogenation of dimethylamineborane.

**Figure 6:**
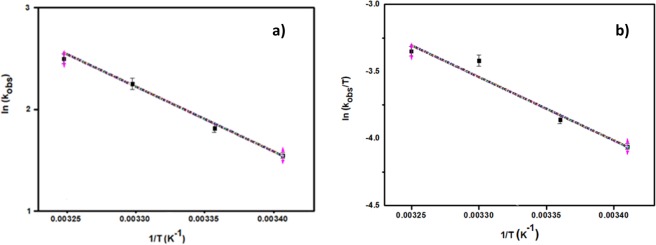
(**a**) Arrhenius parcel at different temperatures, (**b**) Eyring parcel at different temperatures.

The dimethylamine borane dehydrogenation reaction with AuNi@SiO_2_ nanohybrid was performed at different temperatures (20, 25, 30, 35 °C). As displayed in Fig. 5a, at four different temperatures, the rate constants for dehydrogenation of dimethylamine borane were determined. Arrhenius and Eyring graphs obtained with these calculations are given in Fig. 6a,b. As a result of the calculations based on Arrhenius and Eyring graphics; E_a_ = 26.013 kJmol^−1^, ΔH = 23.51 kJmol^−1^, ΔS = −122.83 kJmol^−1^. It has been observed that AuNi@SiO_2_ nanohybrid used in dehydrogenation of dimethylaminborane exhibits very good catalytic performance compared to the literature studies.

NMR data obtained as a result of this study shows that (CH_3_)_2_NHBH_3_ (δ = 712,7 ppm) provides complete conversion to [(CH_3_)_2_NBH_2_]_2_ (δ = ∼5 ppm). Thus, even at room temperature (at 25 °C), it can be seen that the amount of dimethylamine borane dehydrogenation yields 1.0 equivalent H_2_.

The TOF value of AuNi@SiO_2_ nanohybrid, which has a high level of stability for dehydrogenation of dimethylamine borane and its activation parameters is 546.9 h^−1^ as listed in Table 1. This value is one of the best values according to TOF values of catalysts in literature. Namely, the monodisperse AuNi@SiO_2_ nanohybrid prepared for the hydrogen production from the dimethylamine borane has been rapidly converted at room temperature. This indicates that the efficiency of the nanocatalyst obtained is high.

**Table 1:** TOF values of catalysts for dehydrogenation of dimethylamine borane.

Entry	(Pre) Catalysts	Conv. %	TOF (Turnover Frequency)	Ref.
1	AuNi@SiO_2_	100	546.9	This Study
2	Carbon Stabilized Palladium particles	95	2.8	^49^
3	Grafen oxide-based Ruthenium nanoparticles	100	410.01	^50^
4	Rhodium (III) chloride	90	7.9	^49^
5	Iridium (III) chloride	25	0.3	^49^
6	Ruthenium (III) chloride	77	2.7	^49^
7	Vulcan Carbon-based Pt nanoparticles	100	23.14	^26^
8	BA-based Platinum nanomaterials	100	24.8	^18^
9	TBA-based Platinum nanoparticles	100	31.24	^18^
10	Polymer supported Ruthenium-Nickel	100	458.57	^19^
11	Amylamine stabilized Platinum Nanoparticles	100	15.00	^20^
12	Activated Carbon Stabilized Pt Nanoparticles	100	34.14	^21^
13	PVP stabilized Palladium-Cobalt nanoparticles	100	330.00	^22^
14	Grafen oxide-based RuPtNi nanomaterials	100	727.00	^23^
15	Carbon nanotube-based Ruthenium-cobalt nanoparticles	100	775.28	^24^
16	PEDOT supported Palladium Nickel Nanoparticles	100	451.28	^29^
17	Polymer-graphene based Platinum Nanomaterials	100	42.94	^25^
18	Graphene oxide stabilized Palladium-Nickel Nanomaterials	100	271.90	^32^
19	Graphene oxide-based Palladium Nanoparticles	100	38.02	^11^
20	Carbon black hybrid supported platinum nanomaterials	100	70.28	^26^

The very good activation parameters of AuNi@SiO_2_ nanohybrid can be described by Au and Ni synergistic effect on the homogeneous distribution of nanohybrid on a support material, the catalytic activity of Au (0) and Ni (0) percentages, small particle size and the large surface area of nanohybrid.

**Figure 7:**
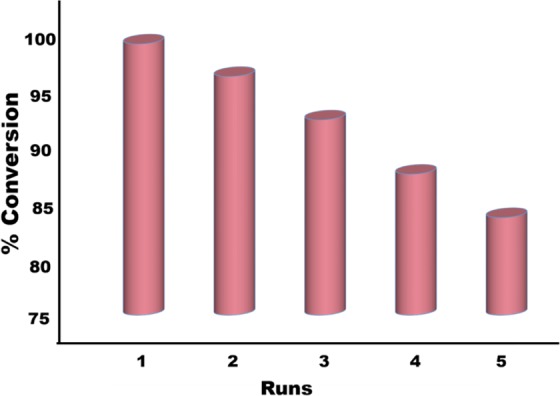
Plots of % conversion versus time for AuNi@SiO_2_ catalyzed dehydrogenation of DMAB at room temperature for 1^st^ and 5^th^ catalytic runs.

The reusability of synthesized nanohybrid is one of the important factors for both cost and easy application. For this purpose, re-usability of the produced AuNi@SiO_2_ nanohybrid was evaluated. As shown in Fig. 7, the AuNi alloy nanohybrid on the SiO_2_ support material maintained its catalytic activity with approximately 84% of its initial performance even at the end of the fifth experiment in the dehydrogenation reaction with DMAB. However, in the ongoing trials, the AuNi@SiO_2_ nanocatalysts decreased their catalytic activity due to increased aggregation and reduced surface area. However, its reusability is tremendously good. AuNi@SiO_2_ nanohybrid, which has a high conversion frequency, showed superior catalytic performance as a high-yield catalyst for the dehydrogenation of dimethylamine borane. The stable and efficient structure of Au and Ni nanohybrid is due to the SiO_2_ support material employed in the preparation of nanohybrid. The increase in surface area of the AuNi alloy due to the silica-based support agent has led to increased efficiency. In summary, according to studies and calculations, the surface area of the expanded AuNi@SiO_2_ nanohybrid with silica provided a very high TOF value (546.9 h^–1^). At the same time, the calculated activation energy value E_a_ = 26.013 kJmol^−1^ is among the lowest activation energies in the literature for DMAB dehydrogenation reaction.

**Figure 8:**
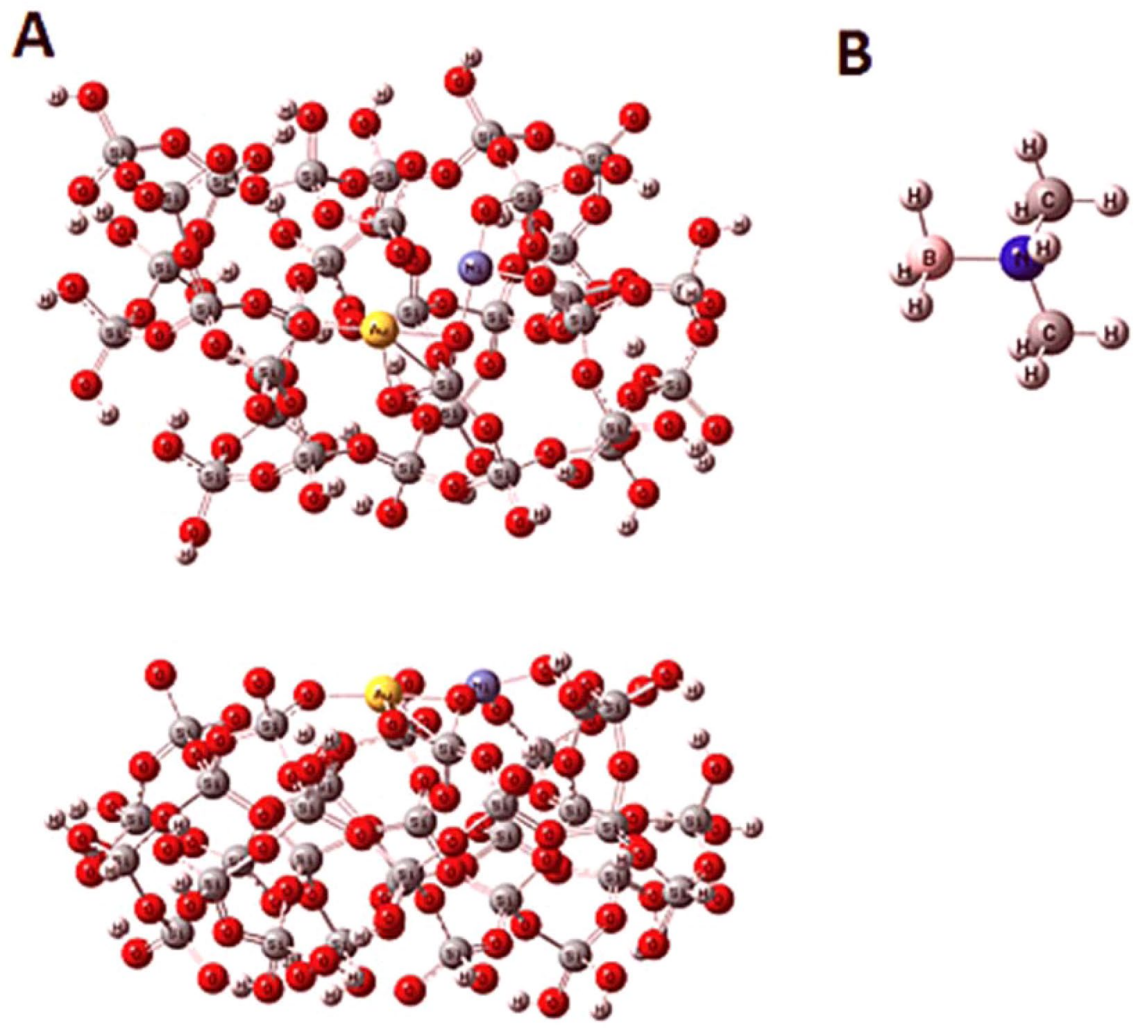
Optimized structures (**A**) AuNi@SiO_2_ cluster (top view and side view) and (**B**) molecule DMAB.

In order to clarify the activity of AuNi@SiO_2_ catalyst, the theoretical calculations have been utilized. For this purpose, initially, SiO_2_ cluster was optimized by EG calculation. The optimized SiO2 cluster structure is shown in the Supporting Information in Fig. S2. Then AuNi@SiO_2_ cluster was optimized geometrically by utilizing EG calculations. For this AuNi@SiO_2_ cluster optimization, neutral charge and doublet SM were used. The minimum SPE energy of the cluster corresponds to the sextet SM. Similarly, the doublet SM was obtained for the structure containing the AuNi@SiO_2_ cluster with a DMAB molecule. Figure 8 indicates EGs for AuNi@SiO_2_ cluster. The charge and SM for the adsorbing molecule (DMAB molecule) have been determined as neutral and singlet, respectively. The DMAB molecule’s optimized geometry has been represented in Fig. 8.

**Figure 9:**
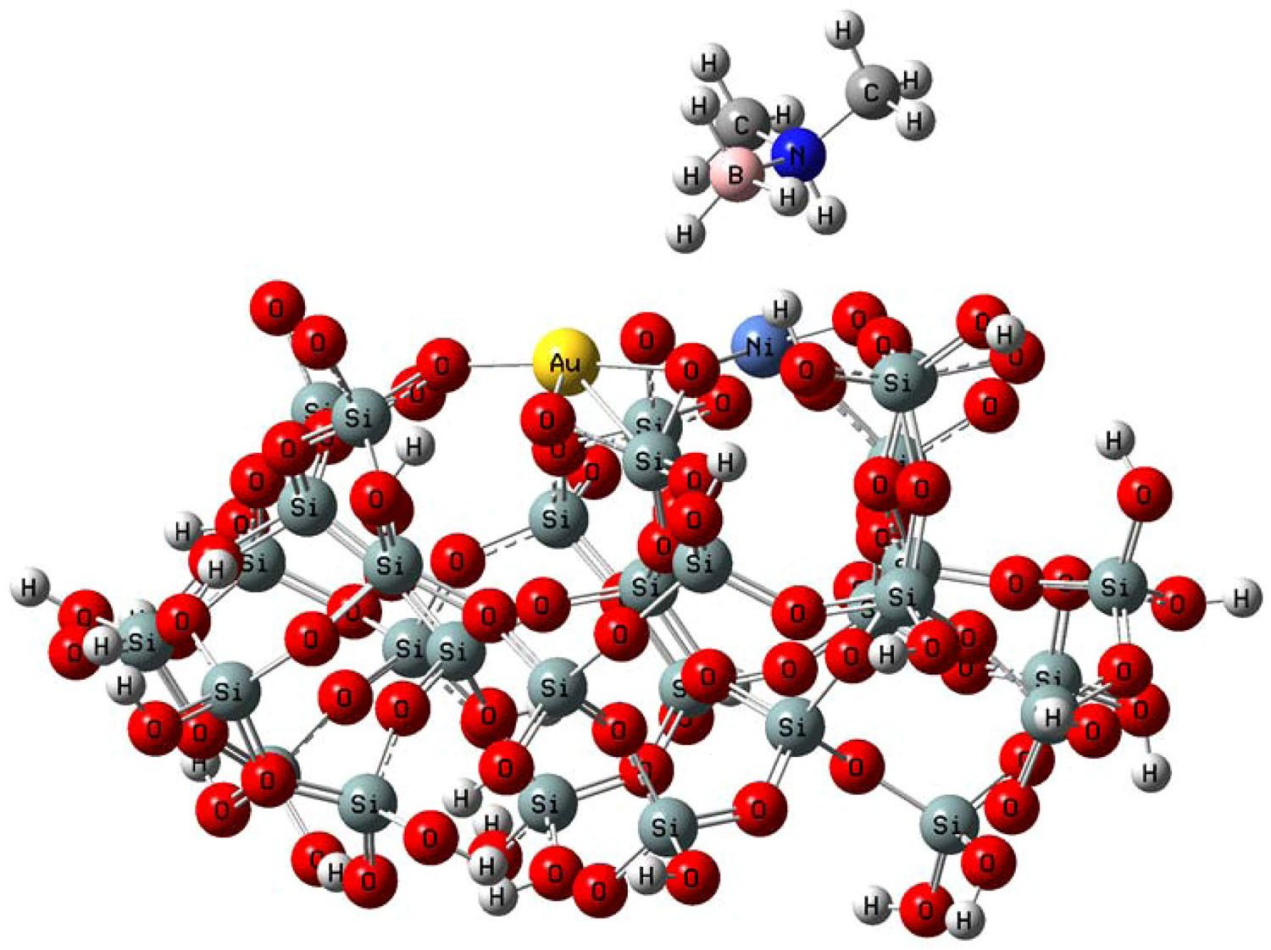
Optimized structure of DMAB molecule adsorbed on AuNi@SiO_2_ cluster.

After obtaining the optimized geometries of cluster and adsorbing molecule (DMAB), adsorption of the DMAB molecule has been examined on AuNi@SiO_2_ cluster by optimization (EG) calculations. Two probabilities according to the position of the DMAB molecule on the catalyst surface for the DMAB adsorption on AuNi@SiO_2_ cluster have been mentioned for EG calculations. These positions have been represented in Fig. S3 in Supporting Information. For these structures, total energy values (containing zero point energy correction) with DMAB adsorption on the AuNi@SiO_2_ cluster were determined to be −15783.831058 a.u. and −15783.903295 a.u. respectively, which suggests that DMAB adsorption is most suitable for configuration 2. For this configuration, optimized geometry for adsorbed DMAB molecule is shown in Fig. 9 on AuNi@SiO_2_ cluster. It can be shown that the DMAB molecule was adsorbed on Ni atom of the AuNi@SiO_2_ cluster.

The adsorption energy (∆E), enthalpy (∆H), and Gibbs free energy (∆G) values for DMAB adsorption have been computed by using equation 8 in Supporting Information. Furthermore, chemical potential, ∆E, and ∆G, chemical hardness, electronegativity, electrophilicity, and HLG values were computed for both α and β molecular orbitals (spin up and spin down, respectively). These values which have been tabulated in Table 2 were calculated by using the HOMO/LUMO values of the optimized AuNi@SiO_2_ cluster-DMAB system^38,39^. For the optimized AuNi@SiO_2_ cluster-DMAB structure, the values were calculated employing equations 4–7 in Supporting Information where ${\rm{I}}\cong -{\epsilon }_{{\rm{HOMO}}}$ and $A\cong -{\epsilon }_{{\rm{LOMO}}}$. In addition, the chemical potential value has been determined by utilizing equation 5 in Supporting Information where ${\rm{I}}\cong -{\epsilon }_{{\rm{HOMO}}}$ of free DMAB and $A\cong -{\epsilon }_{{\rm{LOMO}}}$ of the AuNi@SiO_2_ cluster^21,38–41^.

**Table 2:** Comparison of hydrogen adsorption energy values (Energy values are in units of kcal/mol). The HOMO and LUMO values of optimized SiO2 cluster were calculated to be 162.7 and 62.4  kcal/mol, respectively.

Properties	AuNi@SiO_2_ Cluster		AuNi@SiO_2_ Cluster with adsorbed DMAB	
	α MO (Spin Up)	β MO (Spin down)	α MO (Spin Up)	β MO (Spin Down)
HOMO	−193.0	−193.0	−185.7	−185.7
LUMO	−136.1	−146.7	−149.0	−139.9
Chemical Hardness	28.4	23.2	18.3	22.9
Chemical Potential	−164.6	−169.9	−167.4	−162.8
Electronegativity	164.6	169.9	167.4	162.8
Electrophilicity	476.1	623.2	763.8	578.1
HLG	56.9	46.3	36.7	45.9
∆E	—	—	−56.0	
∆H	—	—	−56.6	
∆G	—	—	−42.3	

Before the DMAB molecule adsorption, the values of chemical potential for α and β molecular orbitals (MOs) have been computed as −151.0 and −156.3 respectively by using equation 5 in Supporting Information where ${\rm{I}}\cong -{\epsilon }_{{\rm{HOMO}}}$ of free DMAB molecule and $A\cong -{\epsilon }_{{\rm{LOMO}}}$ of the AuNi@SiO_2_ cluster^21,38–41^. These values designate that AuNi@SiO_2_ cluster has a high value of chemical potential for the DMAB molecule interaction. AuNi@SiO_2_ cluster’ activity based on electrophilicity, electronegativity, chemical hardness and potential values has been obtained. The chemical potential has been related to the adsorption energy. Then, if a cluster has a low chemical potential, the adsorption energy of DMAB on the cluster is also comparatively low^42,43^. Afterward, the DMAB molecule adsorption on AuNi@SiO_2_ cluster, the chemical hardness, the HOMO-LUMO gap (HLG) and the chemical potential values have reduced as the electronegativity and the electrophilicity values have risen. Additionally, ΔG for DMAB molecule adsorption on AuNi@SiO_2_ cluster has been determined to be −42.3 kcal/mol, which designates DMAB molecule adsorption happens instantaneously on the AuNi@SiO_2_ cluster. The relative energy adsorption value for the adsorbing molecule adsorption has been computed as −56.6 kcal/mol on the AuNi@SiO_2_ cluster meaning DMAB molecule has been strongly adsorbed on it.

**Figure 10:**
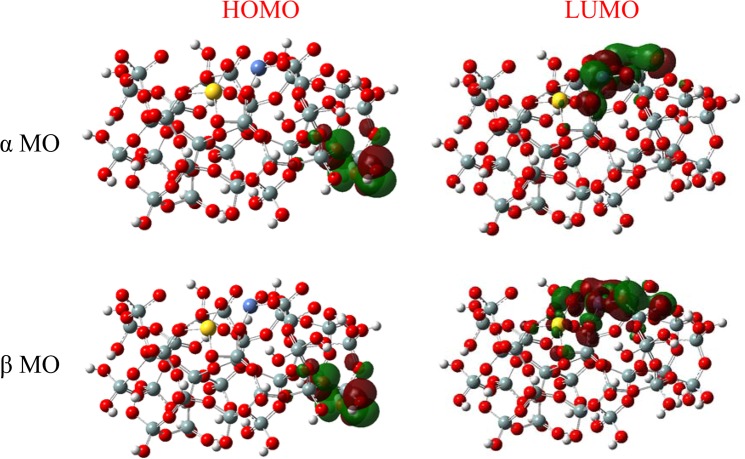
The HOMO/LUMO distributions of α and β MOs for the optimized AuNi@SiO_2_ cluster.

The HLG was widely used as a measure of kinetic stability, and it was commonly recognized that if the gap between HOMO and LUMO is narrow, the kinetic stability is weak and the chemical reactivity is good. In the meantime, it defines energetically opportune for the HOMO losing electrons or gaining electrons of the LUMO. According to the Table 2, it has been obviously stated that the α-HLG and β-HLG values of 56.9 kcal/mol and 46.3 kcal/mol respectively for the AuNi@SiO_2_ cluster is meaningfully smaller than the HOMO-LUMO gap value of 100.4 kcal/mol for the SiO_2_ cluster. After this, it might be clearly concluded that the SiO_2_ cluster’s chemical reactivity was knowingly improved by Au and Ni atoms addition on the SiO_2_ cluster. In other words, the AuNi@SiO_2_ cluster’s chemical reactivity is higher than the chemical reactivity of the SiO_2_ cluster. The HOMO and LUMO distributions of α and β electrons for the AuNi@SiO_2_ cluster are indicated in Fig. 10. It was obviously stated that LUMOs of α and β electrons were located on Ni atom for the AuNi@SiO_2_ cluster. So, this clarifies that the DMAB molecule has been adsorbed on Ni atom of the AuNi@SiO_2_ cluster. The chemical potential values for the AuNi@SiO_2_ cluster have been decreased after the DMAB molecule adsorption meaning the active site of the AuNi@SiO_2_ cluster has still the activity for the other DMAB molecules.

**Figure 11:**
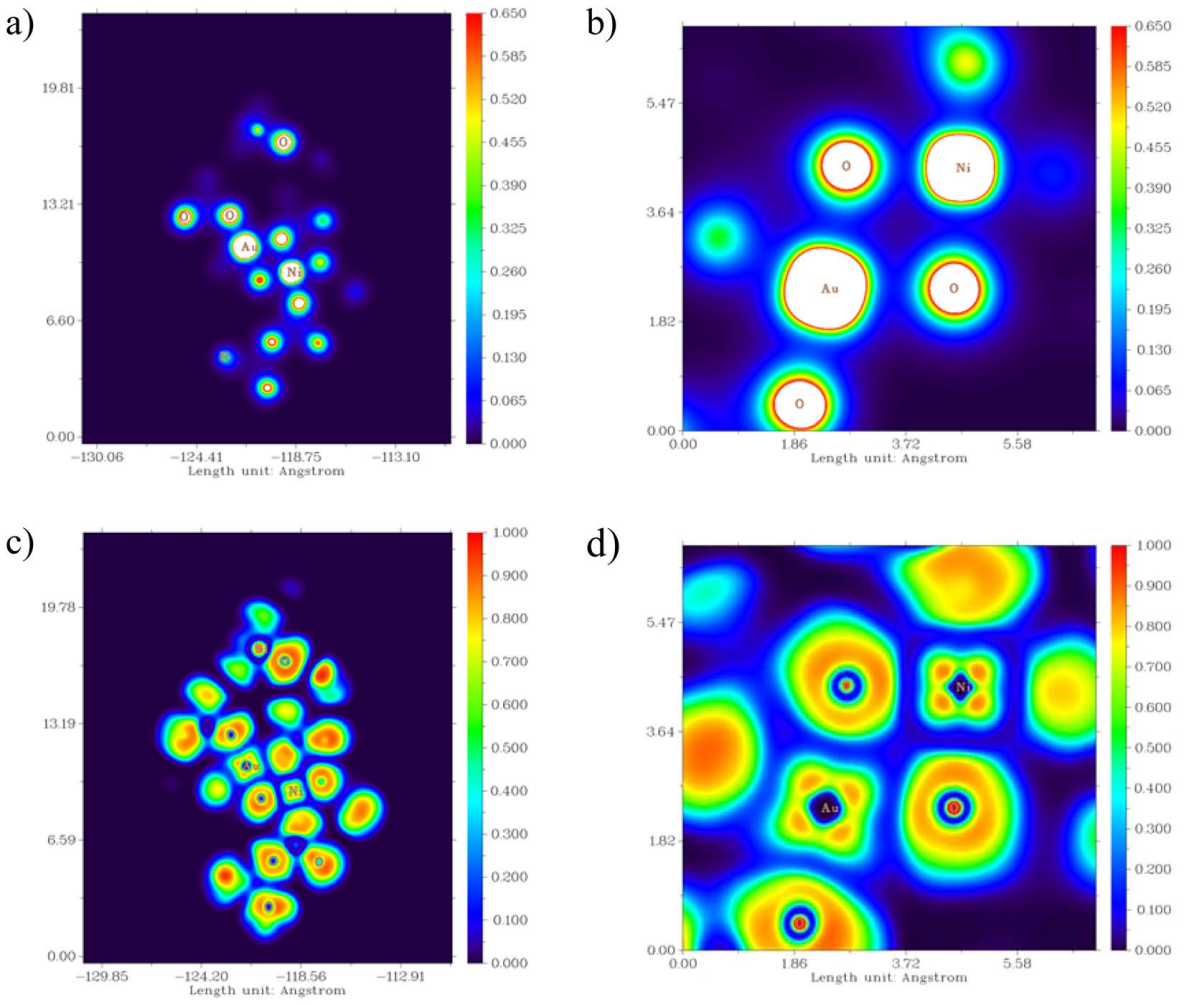
Distribution map of electron density (ED) for optimized AuNi@SiO_2_ cluster; (**a**) at cluster level and (**b**) at Au and Ni atoms level of the cluster, distribution map of electronic localization function (ELF) for optimized AuNi@SiO2 cluster; (**c**) at cluster level and (**d**) at Au and Ni atoms level of the cluster.

**Figure 12:**
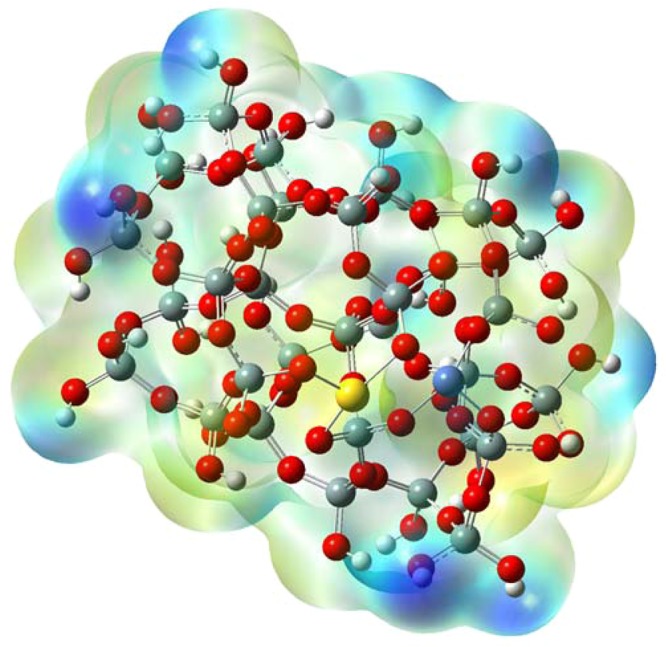
ESP distribution for optimized AuNi@SiO_2_ cluster.

Additionally, Fig. 11a,b show ED distribution map for AuNi@SiO_2_ cluster. Such representations have shown that the densities of electrons are mostly found on the atoms of Au and Ni. ELF distribution map^36,44–46^ has been shown in Fig. 11c,d for AuNi@SiO_2_ cluster. The ELFdistrubition is a valued tool to obtain the place of electron pairs^46^. The role of electronic localization provides understanding of the empirical principle of electron localization, in particular, pair electron localization. Depending on the electronic localization function graph, the atoms in which they exhibited larger values of the electronic localization function in AuNi@SiO_2_ cluster are Au and Ni atoms. Moreover, the negative and positive distribution of electrostatic potential (ESP) regions (Fig. 12) on the surface of the van der Waals was shown between the colors red and blue^47,48^. Analysis of the ESP distribution of AuNi@SiO_2_ cluster data demonstrations that positive regions are located smoothly on Au and Ni atoms (mainly Ni atom). This is consistent with the distribution of charges for Au and Ni atoms (+0.417 and +0.738, respectively) gathered by Mulliken population analysis. It should be also mentioned that some blue regions (positive) of AuNi@SiO_2_ cluster have been located around the surrounding hydrogen atoms. However, it is not appropriate to regard this since these hydrogen atoms have been utilized for saturating atoms for AuNi@SiO_2_ cluster. In addition, the occupation numbers of 5d and 3d orbitals for Au and Ni atoms respectively in optimized AuNi@SiO_2_ cluster have been calculated with respect to NPA analysis and have been listed in Table 3. These results are relatively in consistent with the XPS results that represent the oxidation states of gold and nickel present on SiO_2_ catalysts.

**Table 3:** The computed occupation numbers of 5d and 3d orbitals for Au and Ni atoms respectively in optimized the AuNi@SiO_2_ cluster.

Metal Atom	α and β Molecular Orbitals (Spin Up/Spin Down)	The occupation numbers of 5d and 3d orbitals					Total occupation numbers of 5d and 3d orbitals
		dxy	dxz	dyz	dx^2^y^2^	dz^2^	
Au	α MO	0.760	0.949	0.985	0.867	0.958	9.040
	β MO	0.758	0.949	0.984	0.869	0.959	
Ni	α MO	0.927	0.995	0.997	0.710	0.985	8.267
	β MO	0.830	0.391	0.914	0.573	0.946	

## Conclusions

The present study paves ways for controlled synthesis of AuNi@SiO_2_ nanohybrid with ultrasonic reduction method and towards promising practical applications. It was characterized by XRD, XPS, TEM, and HR-TEM analysis to better understand synthesized nanohybrid while producing a novel AuNi@ SiO_2_ nanohybrid with an efficient, clean and environmentally friendly method. The prepared AuNi@SiO_2_ nanohybrid was successfully applied in dehydrogenation of DMAB reactions. Thanks to Au and Ni, carbon-free and non-porous SiO_2_ support, AuNi@SiO_2_ nanohybrids formed stable and efficient nanostructures. Furthermore, the AuNi alloy on the support material was homogeneously distributed and very tightly bonded. As given previously in Table 1, AuNi@SiO_2_ nanohybrid in the hydrogen production reaction of dimethylamine borane for the production of hydrogen has a high TOF value (546.9 h^−1^), stability and catalytic activity. At the same time, in the dehydrogenation reaction of the obtained nanohybrid with dimethylamine borane, the fact that it continues its activity with approximately 84% of its first performance even in the fifth attempt that is an indicator of its very successful catalytic property. In addition, according to the data obtained from the hydrogen - DMAB graph, it was found that the rate of hydrogen production was the first-order dependent on AuNi@SiO_2_ nanohybrids and DMAB amount. According to the results of the numerical analysis, the activation values were obtained as E_a_ = 26.013 kJmol^−1^, ΔH = 23.51 kJmol^−1^, ΔS = −122.83 kJmol^−1^. When these values are examined, it is noteworthy that the activation energy is very low. As a result, AuNi@SiO_2_ nanohybrids can be considered as a promising catalyst with excellent catalytic performance, simple handling, very good reusability, and high stability for hydrogen production and storage at room temperature. Theoretical data depending on LUMO, HOMO chemical hardness, electronegativity, chemical potential, ∆E, ∆H, and ∆G values as well as ED, ELF and ESP distributions obtained in the theoretical part of the study supports the experimental data which is consequently high activity of the DMAB dehydrogenation catalyst AuNi@SiO_2_.

## Supplementary information

Supplementary Information.
