# Supplementary material for: Use of silica-based homogeneously distributed gold nickel nanohybrid as a stable nanocatalyst for the hydrogen production from the dimethylamine borane
Source: Sci Rep. 2020 Apr 29;10:7215. doi: 10.1038/s41598-020-64221-y (PMC7190821; doi:10.1038/s41598-020-64221-y)
Supplement: Supplementary file 1 — Supplementary Information. [file 41598_2020_64221_MOESM1_ESM.docx]

**Supplementary Information**

**Use of silica-based homogeneously distributed gold nickel nanohybrid as a stable nanocatalyst for the hydrogen production from the dimethylamine borane**

Oznur Alptekin^a^, Betul Sen^b^, Aysun Savk^b^, Umran Ercetin*^a^, Sibel Demiroglu Mustafov^b^, Mehmet Ferdi Fellah^c^, Fatih Sen*^b^

^a^Department of Mechanical Engineering, Faculty of Engineering, Dumlupınar University, Evliya Çelebi Campus,43100 Kütahya, Turkey

^b^Sen Research Group, Department of Biochemistry, Faculty of Art and Science, Dumlupınar University, Evliya Çelebi Campus,43100 Kütahya, Turkey

^c^Department of Chemical Engineering, Bursa Technical University, Mimar Sinan Campus, 16310 Bursa, Turkey

^*^Corresponding authors: [umran.ercetin@dpu.edu.tr](mailto:umran.ercetin@dpu.edu.tr), fatih.sen@dpu.edu.tr

Tel: 90 274 265 20 31 -37 02, 90 274 265 20 31 -41 25

**Materials and Methods**

Sigma-Aldrich supplied superhydride, dimethylamine-borane, AuCl_3_ and NiCl_2_ graphane oxide. THF and water used during this study were provided from Merck and Milli Q-pure machine, respectively. Before washing all glass pieces and other lab materials with large amount of distilled water, they were cleaned with acetone, then dried. About 290 samples were taken from the Au (0) NPs – Ni(0) samples used in the study and the characterization procedures of the samples were carried out. For XPS analysis Kα lines of Mg (1253.6 eV, 10 mA) was used due to understanding of structure of the catalysts containing Au (0) and Ni(0) NPs. TEM analysis was taken by JEOL 200 kV. Panalytical Empyrean diffractometer designing Ultima+theta–theta high resolution goniometer was used to take XRD analysis. Cu K-alpha radiation, k = 1.54056 Å was used to take X-ray analysis. The catalysts based Au (0) and Ni(0) NPs was prepared using Cu double -side tape (SM Inc.). Due to taking XPS value 1s at 284.6 Ev was taken as reference. Raman microprobe (514 nm) was used for raman analysis. 11B-NMR spectra were recorded on a JEOL ECZ500R (11.75 Tesla) spectrometer with an operating frequency of 0.2 Hz. Morphology and EDAX analysis of the catalyst were analyzed by field emission scanning electron microscopy (FESEM) (FEI, NovaNanoSEM 650).

**Computational method**

Equilibrium geometry (EG) calculations were used to optimize geometries and obtain adsorption energies. In present study, energy difference values include zero-point energy (ZPE) corrections. These energies were calculated using the frequency keyword (freq) in Single Point Energy (SPE) calculations. In addition, vibrational frequency, thermal energy, thermal enthalpy and thermal free energy values were calculated by SPE calculations at 298 K and atmospheric pressure in Gaussian software^1^. These energy values were computed as follows:

$E=E_{\mathrm{electronic}} + ZPE + E_{\mathrm{vibrational}} + E_{\mathrm{rotational}} + E_{\mathrm{translational}}$ (1)

$H = E + RT$ (2)

$G = H - TS$ (3)

where E is the sum of the electronic, zero point and thermal energies, H is the sum of the electronic and thermal enthalpies, G is sum of electronic and thermal free energy, S is the Entropy and T is the temperature used for the vibrational frequency calculations. HOMO and LUMO representations and HOMO/LUMO energy values were calculated by full analysis of population. The chemical hardness, electronegativity, electrophilicity and chemical potential values were obtained to have information about the activity of cluster by using the following equations. ϵ_HOMO_ is the highest occupied molecular orbital energy and ϵ_LUMO_ is the lowest unoccupied molecular orbital energy. These equations based on the Koopman’s approach ^2–5^ are given as follows.

$Chemical hardness (\eta)=\frac{I-A}{2}$ (4)

$Chemical potential (\mu) = -\frac{I+A}{2}$ (5)

$Electronegativity (\lambda) = - \mu$ (6)

$Electrophilicity (\omega) = \frac{\mu^{2}}{2\eta}$ (7)

where ${I\cong-\epsilon_{HOMO} and A\cong-\epsilon}_{LUMO}$

The theoretical methodology utilized here is given in Supporting Information. The electron density (ED) and electron localization function (ELF) distribution maps have been obtained by using Multiwfn software^6^. Electrostatic potential (ESP) distribution has been obtained by Gaussian software. Moreover, Mulliken atomic charges of atoms were obtained by Mulliken population analysis ^2^.

The convergence criteria are 12x10^-4^ for gradients of root-mean-square (rms) displacement, 18x10^-4^ for max displacement, 3x10^-4^ for rms force and 45x10^-5^ for max force for theoretical calculations utilized in this study. The theoretical methodology utilized here: Firstly, the Spin Multiplicity (SM) for the system including adsorbing molecule and the cluster has been determined by SPE calculations. SPEs were calculated for different numbers of SM for separately system, and then the number of SM which gives the lowest energy based on SPE calculation was accepted to be final SM number for the related system. And then, the adsorbing molecule (DMAB molecule) and the cluster were structurally optimized by EG calculations. Following equation has been used in order to compute the relative energy values for calculations.

$\Delta\left( E/H/G \right)= \left( E/H/G \right)_{System}-\left( E/H/G \right)_{Adsortive}-{(E/H/G)}_{Cluster}$ (8)

Here, (E/H/G) _System_ is the calculated energy/enthalpy/Gibbss free energy for the optimized system which contains the geometries of the adsorbing molecule and the cluster, (E/H/G) _Adsorptive_ is the calculated energy/enthalpy/Gibbss for the adsorbing molecule, e.g. DMAB molecule and (E/H/G) _Cluster_ is the calculated energy/enthalpy/Gibbss for the original cluster.

| 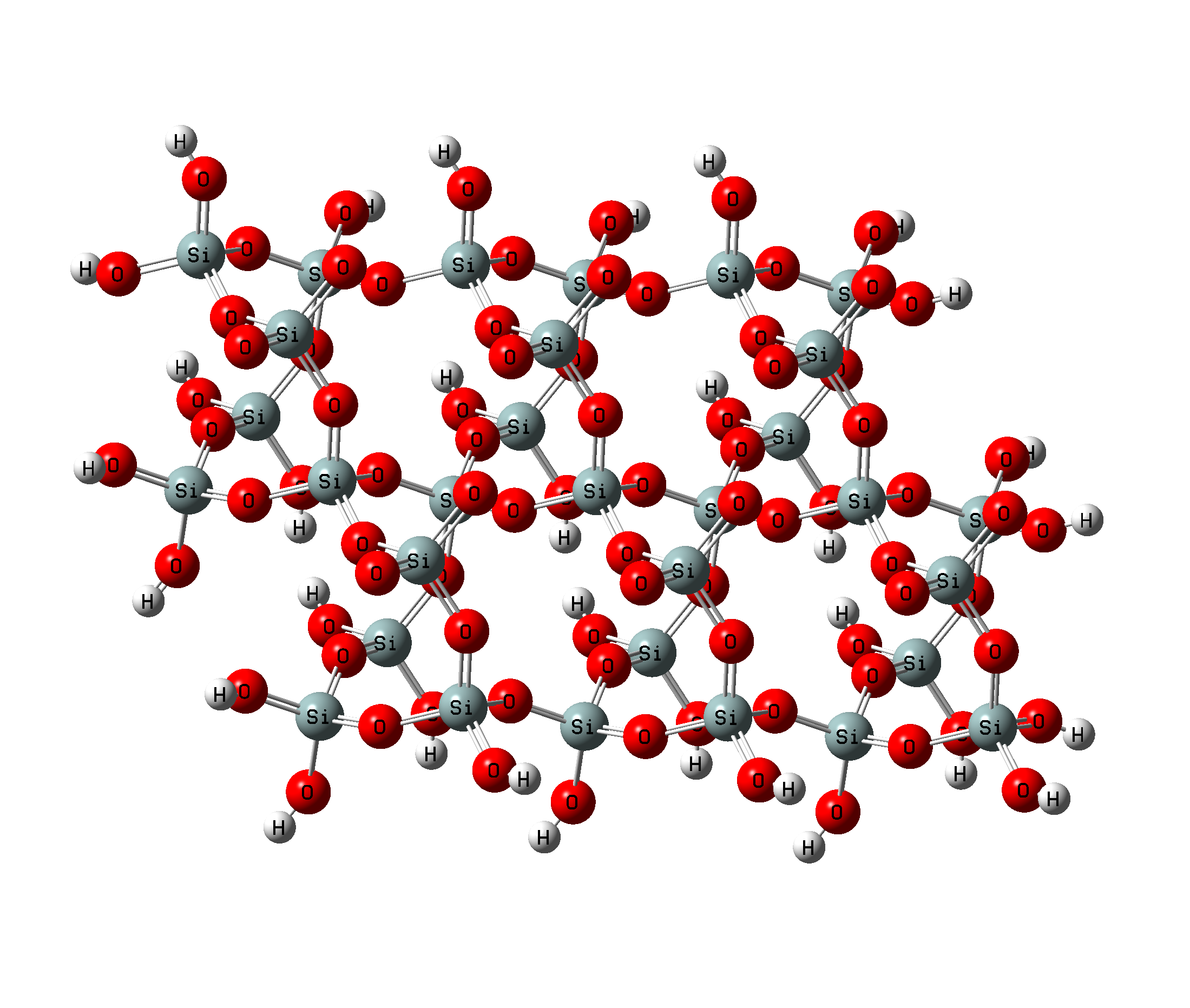 |
| --- |
| Figure S1. The structure of SiO_2_ (002) cluster |

| a) | 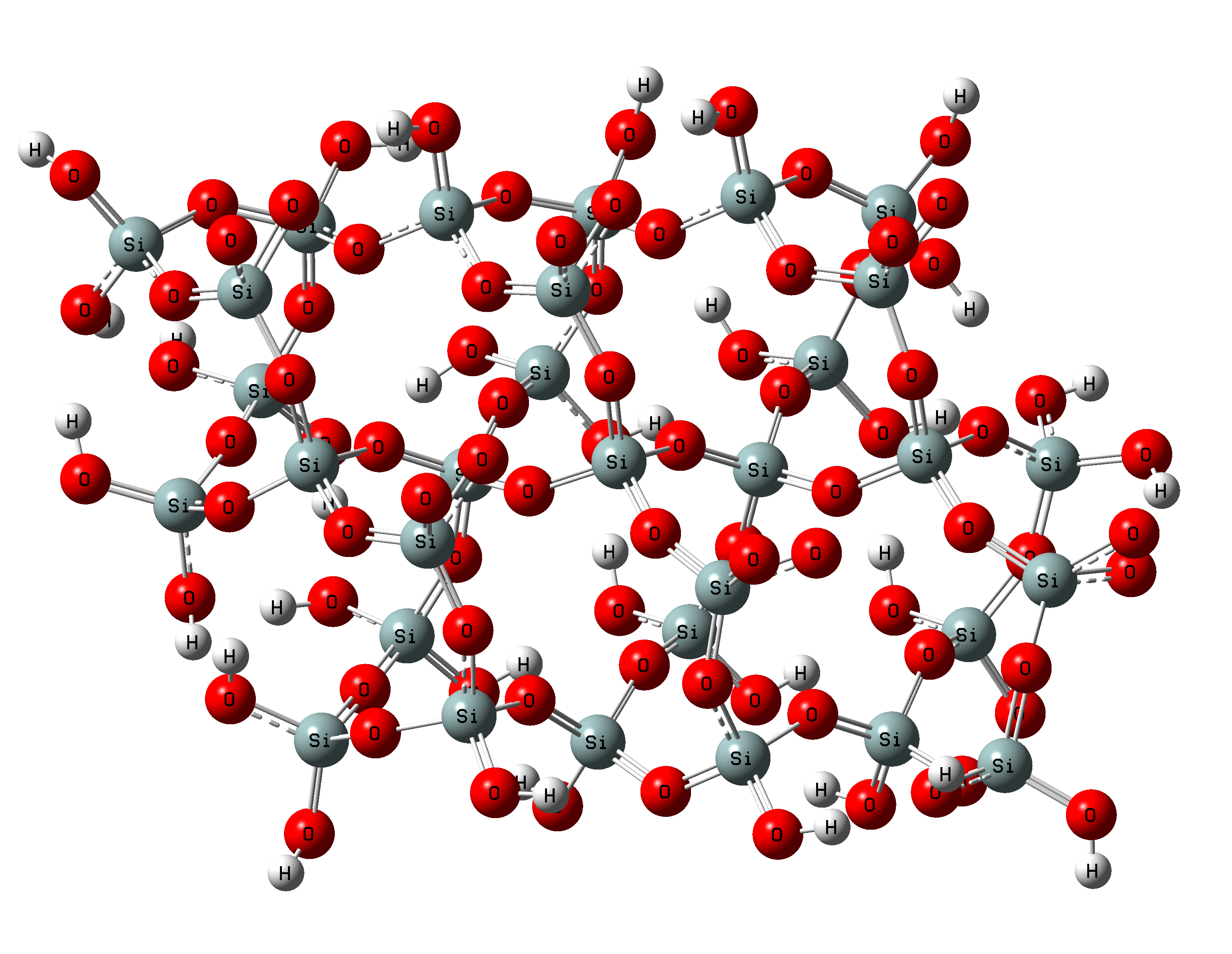 |
| --- | --- |
| b) | 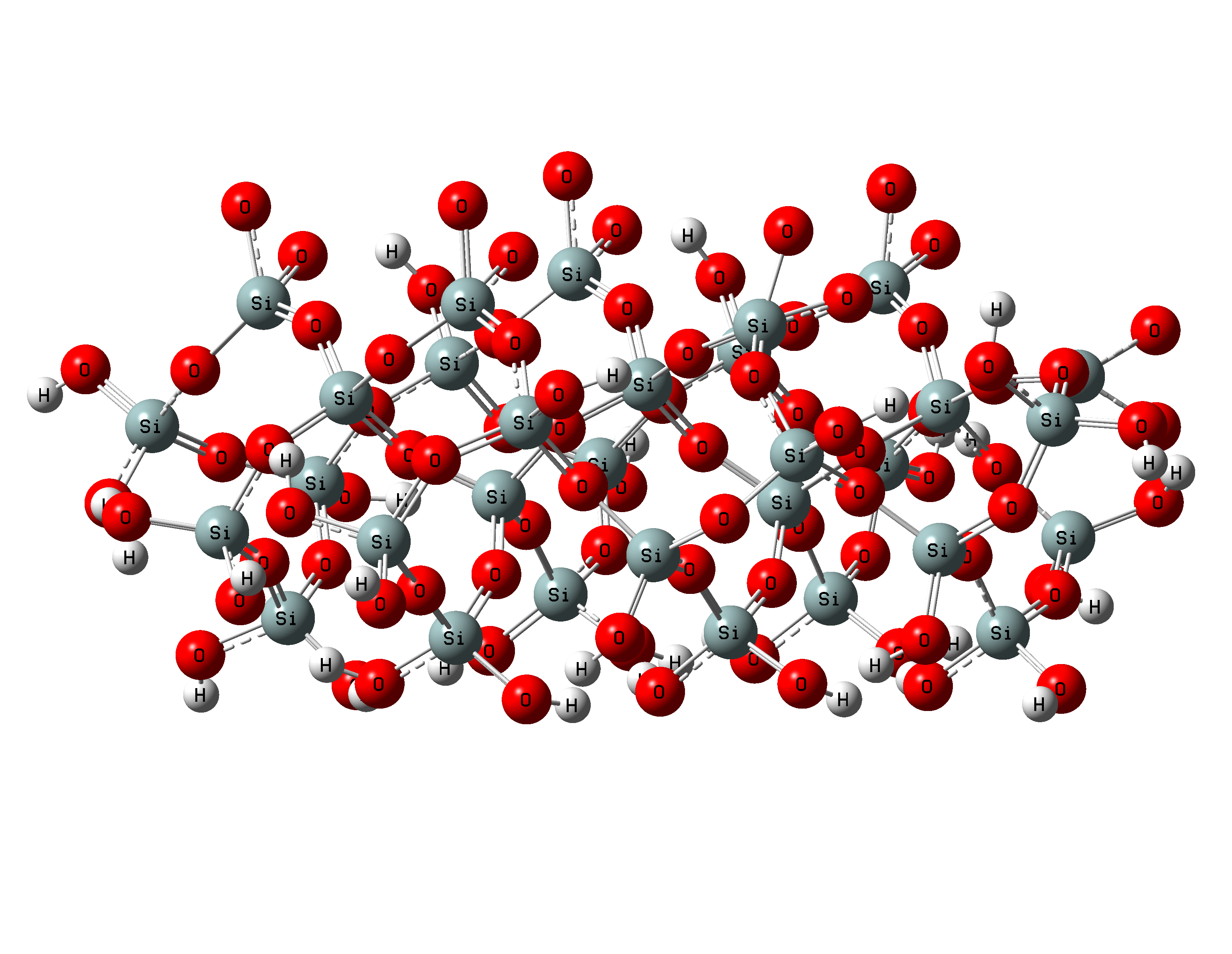 |
| Figure S2. The optimized structure of SiO_2_ (002) cluster | |

| a) | 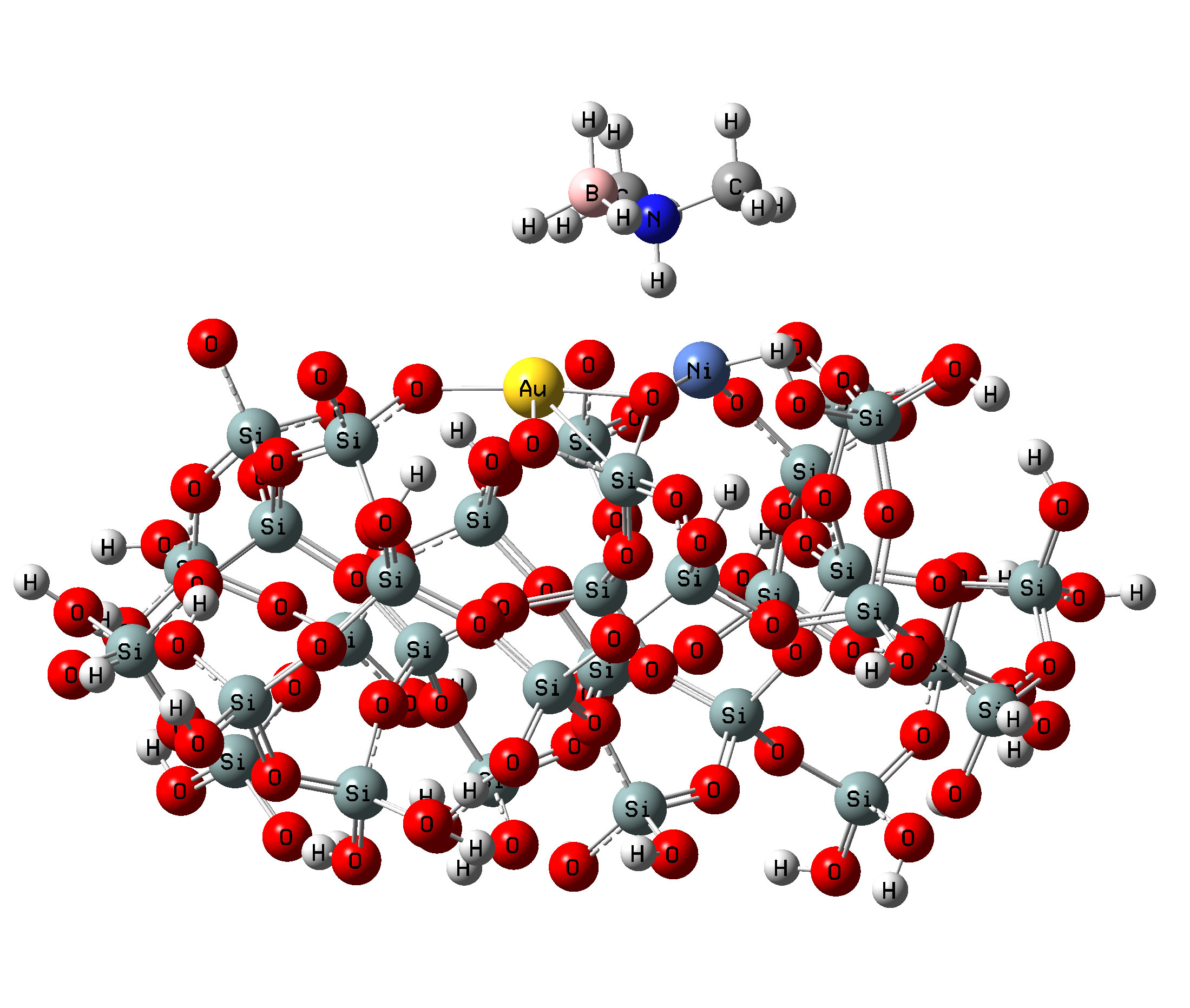 |
| --- | --- |
| b) | 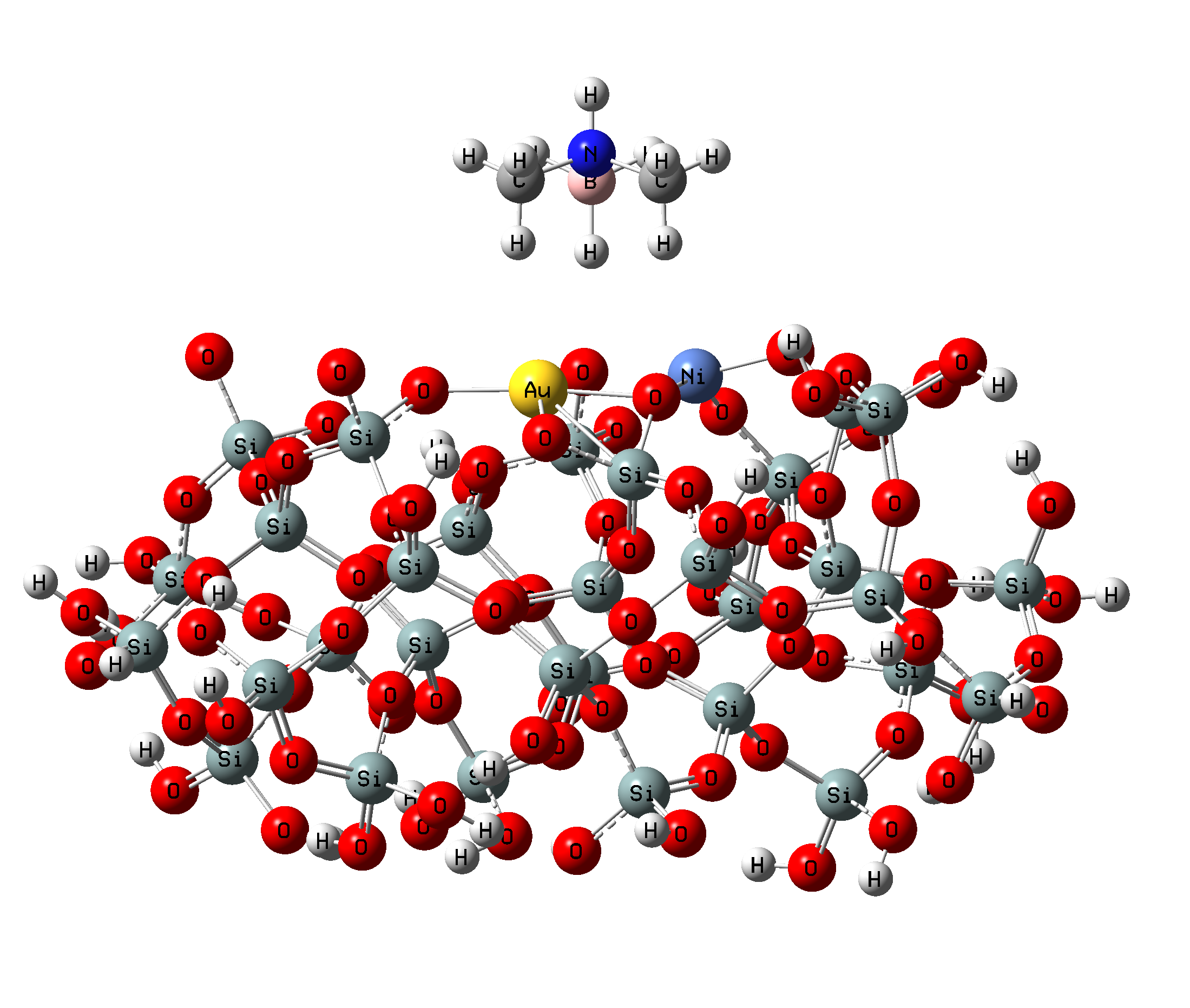 |
| Figure S3. Two possibilities for the adsorption of DMAB on AuNi@SiO_2_ cluster a) Configuration 1 b) Configuration 2 | |


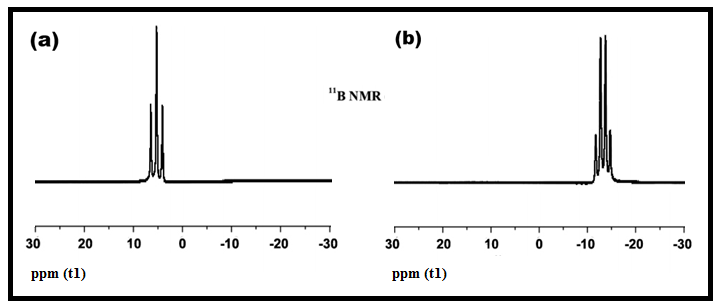


Figure S4: 11B-NMR spectra of a) the DMAB and the catalytic dehydrogenation of DMAB in the presence of AuNi@SiO_2_ at room temperature


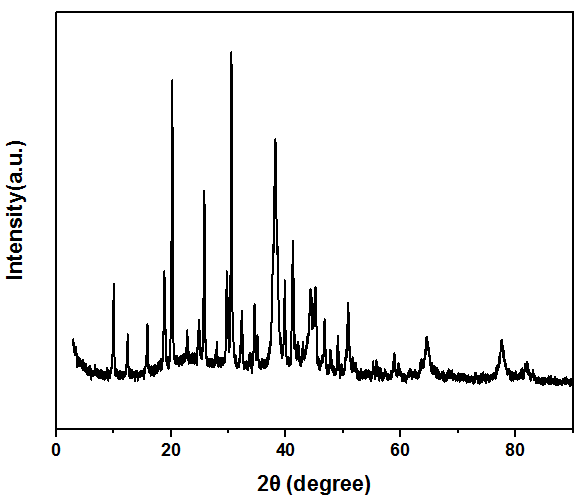


Figure S5: X-ray diffraction pattern of catalysts for H_2_ production (afteruse)


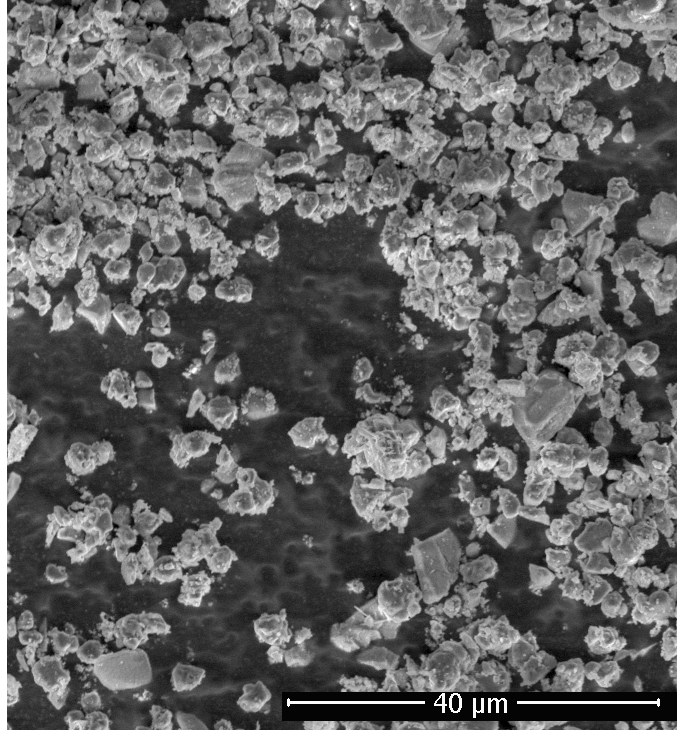


Figure S6: SEM images of catalysts for H_2_ production (afteruse)


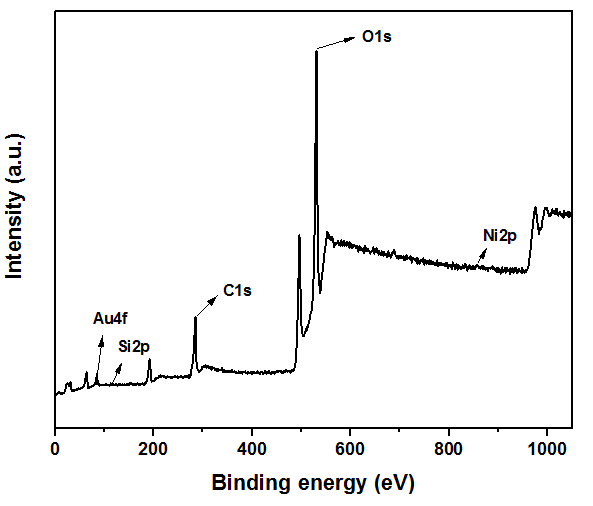


Figure S7: Survey spectra of AuNi@SiO_2_ nanohybrids


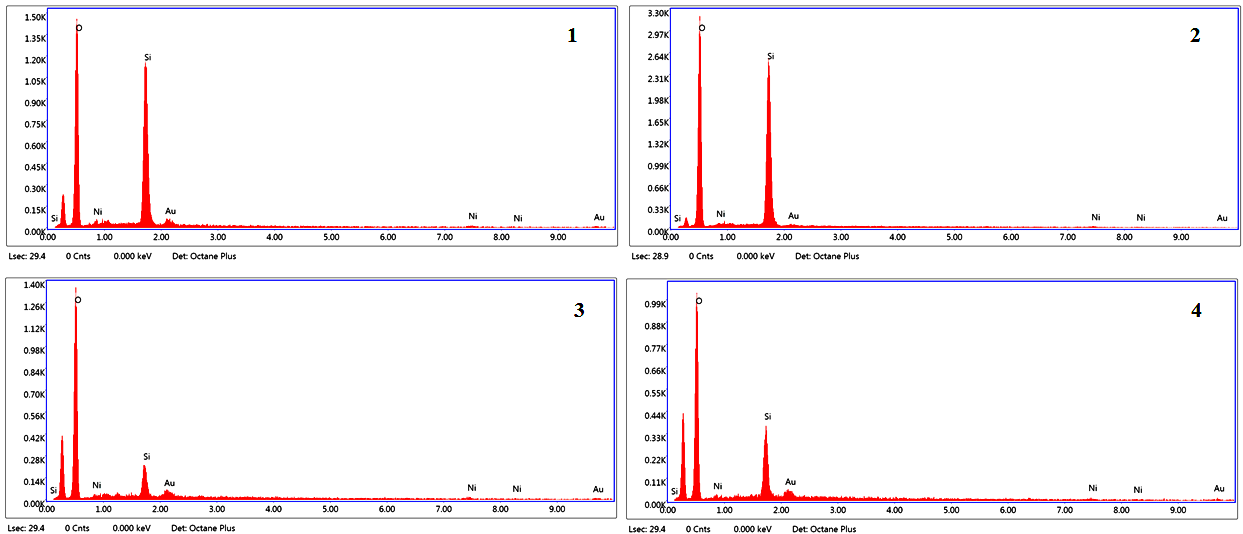


Figure S8: The results of EDX analysis at four different areas

| Table S1. Cartesian coordinates of the optimized geometry of AuNi@SiO_2_ cluster |
| --- |
| O -118.80685896 2.66713008 7.13809033  O -122.16975868 8.04490397 5.42654319  O -123.91115559 11.82466058 6.23161436  O -116.70158390 7.43768752 5.58669959  O -120.20034300 13.04522510 5.91485468  O -121.67931297 16.66553595 6.32812105  O -119.30980754 4.82175722 8.66563190  O -122.20537125 9.49159492 7.72628576  O -125.27694990 13.89368472 7.17609488  O -114.59693273 5.17482856 9.39204054  O -117.39687041 9.53204460 7.18083912  O -120.44340258 14.16998245 8.35601722  O -123.25013066 17.49458334 8.23653439  O -112.42333401 8.54646261 8.02192672  O -115.94889858 14.69628542 8.47775085  O -118.10957745 16.74218251 8.51137450  O -121.44545050 3.34711760 8.30429120  O -124.79082598 9.18707691 7.07858858  O -127.78909506 13.44061142 7.73827422  O -116.79482916 4.10963207 8.20259217  O -119.89732288 8.91994866 6.53775250  O -122.99763320 13.35243329 8.30770439  O -125.81667789 17.66399049 9.15516610  O -114.99351563 8.35713183 7.33379251  O -118.05090016 13.33821784 7.55287067  O -120.71476679 17.06341766 8.83255430  O -118.24748315 5.11018306 6.17380476  O -121.57754531 10.67139994 5.39730313  O -122.89883475 14.23114756 5.77838939  O -115.67779632 9.88772918 5.18300308  O -119.21050372 15.46633971 6.34386232  O -121.31567295 19.16620130 7.26522230  Si -118.29255741 4.17416017 7.50363602  Si -121.47853254 9.26269888 6.26507355  Si -123.77198886 13.33835813 6.85001724  Si -116.18192708 8.82443444 6.32785672  Si -119.46519356 13.99473816 7.05055711  Si -121.72603827 17.63806092 7.65496886  O -120.36149388 2.81326631 13.11173935  O -122.55528389 4.40386419 12.65948796  O -120.75612252 4.12575086 10.79143512  O -123.73363674 7.71540508 9.01795726  O -125.86188285 12.26055854 9.23913388  O -116.51073189 3.83860341 10.83376115  O -118.81466327 7.85951402 8.73741394  O -121.21486627 12.09222445 9.81004321  O -123.96298851 16.36289191 10.54048974  O -114.23006791 7.96213006 9.92121605  O -116.26752308 12.04325047 9.03111822  O -119.32883093 15.99987966 10.84556704  O -121.29667126 20.13841405 9.97969759  Si -120.92970271 4.17145396 12.40128799  Si -122.98166158 6.60590957 9.93591357  Si -125.02290619 11.32192965 10.27726841  Si -117.95554242 7.42601276 10.05142593  Si -120.18822590 10.85905635 10.00636543  Si -122.90901824 15.48933040 11.43090481  Si -114.99980913 11.41741746 9.86332797  Si -118.39327572 14.76820252 11.42280644  Si -120.09088374 19.64682670 10.98878701  O -120.11102793 5.42001738 13.10831594  O -122.32321215 7.34308073 11.28990728  O -124.08923009 12.29284004 11.22768091  O -117.22860263 8.79068701 10.59208288  O -119.11908518 11.26714943 11.18130476  O -121.96234766 16.47306445 12.31546935  O -114.08867227 12.73348551 10.24981030  O -117.12244862 15.38530468 12.24619534  O -119.17114458 20.90910462 11.45863878  O -118.57186140 7.63796148 13.79259060  O -123.05253164 9.73831217 12.39885980  O -125.08762986 12.51354109 13.74442848  O -117.38000077 9.94216506 13.01638621  O -119.57438558 11.27456585 13.77137943  O -121.46902958 17.75864649 14.61076631  O -117.50532053 5.36112895 13.04217778  O -120.79611456 8.94916231 13.08197741  O -122.49203451 12.59627356 13.50374729  O -115.44428733 8.25849440 12.63317086  O -117.19592435 12.29803736 12.92818020  O -119.44542085 16.75798819 13.39300817  O -121.75094770 5.92163127 9.09190243  O -124.02144195 10.33097090 9.44841284  O -126.35452571 14.89893957 9.45759973  O -116.84397756 6.30694485 9.73448675  O -119.38124065 10.49404539 8.64379979  O -121.99965571 14.53604669 10.47590678  O -123.82736249 19.06734528 10.26412322  O -114.20604485 10.38386047 8.88793195  O -117.83388139 13.89721132 10.16493842  O -119.07075860 18.58349783 10.25834249  O -124.04335254 5.48575675 10.45735134  O -126.11451578 10.50144652 11.17741695  O -118.97413030 6.89670999 11.23560851  O -121.02963360 9.53082712 10.49183586  O -123.83369092 14.59412384 12.44288423  O -115.45509207 10.63547944 11.23483618  O -119.28974167 13.70000114 12.32953547  O -120.79221558 18.86884173 12.26984486  Si -118.82347830 6.37023313 12.78103627  Si -121.84004739 8.81584965 11.70762440  Si -123.87930547 12.97012437 12.69854749  Si -116.33926894 9.40173703 11.81691856  Si -118.79663684 12.13758044 12.54036249  Si -120.91435075 17.45185651 13.05614340  Si -120.81619260 4.57696771 9.21161020  Si -123.71358621 9.19944357 8.31404463  Si -126.31436856 13.60322473 8.41658928  Si -116.20880716 4.80730989 9.55561246  Si -118.87231977 9.19013201 7.77972631  Si -121.67597651 13.54257932 9.22635742  Si -124.23675306 17.63176482 9.52171548  Si -113.95944243 8.79158370 8.54048624  Si -117.01469067 13.47605280 8.81757504  Si -119.29101487 17.09383159 9.61117633  Au -121.63386829 10.82104994 13.37823962  Ni -118.99664997 9.36075156 13.53247103  H -120.48636171 1.98554686 12.63432722  H -119.75926900 2.53558284 7.25388966  H -123.13447791 8.00222392 5.45679414  H -117.12590044 7.57116047 4.72980520  H -117.64201813 5.87097694 6.14113923  H -121.02035183 13.46329063 5.57788894  H -120.87971398 11.33770601 5.55569017  H -123.10494188 11.46665285 5.79372304  H -118.68168949 16.06659928 6.91013982  H -120.77652812 16.35845921 6.09566639  H -122.69344783 15.17068124 5.97950511  H -121.22180696 19.75835832 8.03320036  H -122.21072071 19.83691844 10.17938935  H -124.52034444 19.46397511 10.80579810  H -125.74973167 10.06007269 11.95497106  H -123.74752764 5.01977932 11.26183358  H -122.82181988 4.58145904 13.56966918  H -116.62395360 4.27690885 11.69406440  H -112.26971809 8.65954392 7.07768294  H -115.96126863 10.79979067 5.31668621  H -115.10256054 14.60723855 8.94144215  H -117.21995006 16.43067650 8.74855196  H -113.50233542 12.66017700 11.01120190  H -117.38256199 15.95705472 12.98319108  H -128.52456042 13.26215290 8.33392433  H -124.88252288 10.07065538 6.67586411  H -122.39780285 3.34711062 8.15727221  H -119.59878330 21.77120246 11.48515892  H -114.27350225 6.98945788 9.83662299  H -113.96532053 4.44774553 9.41538303  H -125.67967019 14.92997396 10.15066222  H -126.24601844 16.78868883 9.09353901 |

| Table S2. Cartesian coordinates of the optimized geometry of DMAB adsorption on AuNi@SiO_2_ cluster (Configuration 2) |
| --- |
| O -118.87172498 2.71978395 7.52909485  O -122.18587901 8.07196016 5.53113180  O -123.96233021 11.88841352 6.26557293  O -116.78193470 7.38517610 5.68884239  O -120.28878704 13.12090173 5.74653048  O -121.76535084 16.75579375 6.08745679  O -119.31736260 4.92467982 9.00271775  O -122.19010237 9.58108938 7.79310952  O -125.31756955 13.96361232 7.20782356  O -114.60129898 5.40047074 9.48603078  O -117.38999958 9.56715725 7.20076830  O -120.43704345 14.33470538 8.15273579  O -123.24505320 17.66945700 8.02913607  O -112.41799461 8.63900047 7.99145743  O -115.95324895 14.86013553 8.10330317  O -118.08547977 16.92461732 8.11949457  O -121.44538488 3.41212497 8.82165118  O -124.79852393 9.27544893 7.23633700  O -127.78812191 13.75036680 7.99246816  O -116.82111498 4.21133708 8.46013766  O -119.90626385 9.01020370 6.56895559  O -122.98110488 13.49213476 8.24647435  O -125.68816455 17.72990804 9.24586697  O -114.96898456 8.44114729 7.25506274  O -118.07920585 13.47098609 7.28140227  O -120.67568070 17.25016611 8.51804584  O -118.35963804 5.12958807 6.46041012  O -121.64100319 10.70534333 5.41614825  O -123.00868825 14.29334600 5.68928679  O -115.82703753 9.80470857 5.05294558  O -119.28582734 15.55883079 6.04879949  O -121.34465176 19.29055541 6.90401004  Si -118.34924182 4.23878164 7.82220570  Si -121.49905245 9.32435242 6.32017328  Si -123.81880146 13.42409527 6.82657903  Si -116.23200047 8.82071422 6.30255956  Si -119.51340690 14.11441858 6.81577635  Si -121.74817957 17.78209021 7.37479814  O -120.35692708 3.28338206 13.67954299  O -122.52437306 4.85180742 13.03320611  O -120.67950030 4.37771312 11.22434979  O -123.68712206 7.85397724 9.18373182  O -125.87569808 12.30995612 9.27430051  O -116.41017975 4.11786836 11.09082048  O -118.85791716 8.01253382 8.82367947  O -121.13698673 12.33503113 9.74676118  O -123.47870295 16.98006330 10.57596811  O -114.27330910 8.20442953 9.88513261  O -116.24765407 12.22333012 8.74330839  O -119.21941031 16.26351280 10.52060564  O -121.21507001 20.37488325 9.60246465  Si -120.92029658 4.55808230 12.81837412  Si -122.95661297 6.82117962 10.20465269  Si -124.96811677 11.46875083 10.34427050  Si -117.97383078 7.62717489 10.13546668  Si -120.11683883 11.10181271 9.97114741  Si -122.68575836 15.79935212 11.34256666  Si -114.99053424 11.63563513 9.61816671  Si -118.26124229 15.05297767 11.10318122  Si -120.04378856 19.82831497 10.61915035  O -120.03195086 5.83951561 13.41891102  O -122.26269067 7.67766961 11.46930551  O -124.06982939 12.55621442 11.19877089  O -117.24019691 9.00912286 10.61928789  O -119.00867129 11.55910536 11.09194340  O -121.61651498 16.45149582 12.39454791  O -114.07925451 12.96544814 9.95164638  O -116.93380116 15.68898427 11.81489974  O -119.15794108 21.05917188 11.22311606  O -118.47165745 8.06143218 13.86202283  O -122.95506460 10.14069406 12.45913219  O -124.96392515 12.96383319 13.72478346  O -117.26167568 10.31630052 12.95911219  O -119.43290475 11.71079793 13.67903133  O -121.02697554 18.12680098 14.37471849  O -117.42522109 5.74737584 13.21884376  O -120.69819825 9.37097257 13.15654135  O -122.36688991 13.05206276 13.36095252  O -115.30103270 8.64677710 12.56145692  O -117.04745997 12.64544031 12.75176081  O -119.05226756 17.21182457 13.05183341  O -121.74521142 6.03732614 9.42320215  O -123.93847172 10.48170302 9.54632169  O -126.03550062 14.96827025 9.63110102  O -116.86325601 6.50015064 9.84749552  O -119.35075898 10.65622208 8.60869745  O -121.92100251 14.80704317 10.30304410  O -123.80870317 19.53076722 9.81697956  O -114.19324511 10.55193532 8.70146598  O -117.78782657 14.10196010 9.86789202  O -118.99444806 18.82586545 9.84000337  O -124.03770569 5.78449768 10.83556030  O -125.99499580 10.65614572 11.32148885  O -118.96792834 7.15082630 11.36508802  O -120.95288014 9.80787459 10.54861494  O -123.80605570 14.94042606 12.18850936  O -115.45950753 10.92154869 11.02108228  O -119.12446480 14.05523894 12.11609526  O -120.77712874 18.94281495 11.81179046  Si -118.75308291 6.73875767 12.93297317  Si -121.76622981 9.16269602 11.80177382  Si -123.79817890 13.34313443 12.60123666  Si -116.27860088 9.71295469 11.73057445  Si -118.65693299 12.49894484 12.39699324  Si -120.62865060 17.66582505 12.80812745  Si -120.80267001 4.70943187 9.61923290  Si -123.68118733 9.31201376 8.43316211  Si -126.25101334 13.72725414 8.53709829  Si -116.19603600 5.00616154 9.73923527  Si -118.87908160 9.30080176 7.80395173  Si -121.63013336 13.75033616 9.10279035  Si -124.08848977 17.94172891 9.39832505  Si -113.96061403 8.93884151 8.45761391  Si -117.00584124 13.64810303 8.50854695  Si -119.22840950 17.31471356 9.24635768  Au -121.51146642 11.26645325 13.34419157  Ni -118.87760255 9.78034478 13.51167098  H -120.72084386 2.41656037 13.46757866  H -119.80679070 2.57637366 7.73746368  H -123.14901864 8.01429614 5.57300459  H -117.22755698 7.45655729 4.83518642  H -117.73947352 5.87353011 6.37147135  H -121.12340857 13.51967092 5.42381137  H -120.94538858 11.38491926 5.51801942  H -123.16534323 11.51517597 5.82449343  H -118.73437967 16.17946400 6.56953536  H -120.87331199 16.44356197 5.82532953  H -122.80134337 15.24019407 5.84833688  H -121.20613538 19.91238631 7.64025472  H -122.15549577 20.16305064 9.80187583  H -124.52719888 19.96503373 10.29176924  H -125.58265507 10.23666273 12.08779619  H -123.73506128 5.38121186 11.67353909  H -122.78396096 5.09803651 13.94889855  H -116.53482321 4.60718911 11.92083267  H -112.24878489 8.66213456 7.04338750  H -116.08341544 10.72881588 5.15436256  H -115.09992841 14.79444743 8.55791998  H -117.19198039 16.60896743 8.33602734  H -113.52358559 12.93792013 10.73864620  H -117.14294048 16.30606230 12.53213600  H -128.48556376 13.61938554 8.64349011  H -124.90569608 10.14791060 6.81378431  H -122.40387305 3.37772877 8.72789600  H -119.54045738 21.93981045 11.15354757  H -114.30784691 7.22749022 9.86019254  H -113.95075281 4.69242914 9.54801543  H -125.47540725 14.78848916 10.39933224  H -125.99704266 16.80418198 9.31825418  C -120.92821995 7.85148502 16.78473609  H -119.86875789 8.07866177 16.93891611  H -121.35751870 8.53730731 16.05364761  H -121.47184268 7.95148143 17.72559895  N -121.10078571 6.46374113 16.28430047  C -120.46524278 5.47239100 17.19196629  H -120.52386292 4.48735135 16.72864547  H -119.41901914 5.73276770 17.37888625  H -121.01949245 5.45999561 18.13214212  B -122.67016086 6.12672306 15.99644196  H -123.26090312 6.25791182 17.04093810  H -123.03434858 6.90682907 15.14132158  H -122.70124738 4.96219878 15.62920343  H -120.62123735 6.40174198 15.38262869 |

Table S3: The results of XPS analysis for chemical composition of AuNi@SiO_2_ nanohybrids

| ***Chemical Composition of Sample*** | ***Spectral Region*** | ***Details*** |  |
| --- | --- | --- | --- |
| Si carbide | Si2p | SiC | Si <2 At. % |
|  | C1s | carbide |  |
| Ni oxide | O1s | Metal oxide |  |
|  | Ni2p | NiO | Ni <2 At. % |
| Si oxide | Si2p | SiO2 | Si <2 At. % |
|  | O1s | SiO2 |  |
| silicate | Si2p | Metal SiO4 | Si < 2 At.% |
| Elemental Ni | Ni2p | Element | Ni < 2 At.% |
| Elemental Au | Au4f | Element | Au < 2 At.% |
| Elemental Si | Si2p | Element | Si < 2 At.% |

Table S4: Elemental ID and Quantification

| ***Name*** | ***Peak BE*** | ***FWHM eV*** | ***Area (P) CPS.eV*** | ***Weight %*** | ***Q*** |
| --- | --- | --- | --- | --- | --- |
| Au4f | 84.60 | 4.27 | 172.27 | 0.33 | 1 |
| Si2p | 101.25 | 5.70 | 189.86 | 1.22 | 1 |
| Ni2p | 854.17 | 17.72 | 1274.78 | 1.34 | 1 |

Tablo S5: The elemental mapping of the catalyst

| **Selected Area** | **Element** | **Weight %** | **Atomic %** |
| --- | --- | --- | --- |
| 1 | Ni  Au | 20.00  20.00 | 10.40  3.10 |
| 2 | Ni  Au | 0.15  0.00 | 0.05  0.00 |
| 3 | Ni  Au | 0.08  5.21 | 0.03  0.51 |
| 4 | Ni  Au | 0.02  4.88 | 0.01  0.49 |

**References**

1. Foresman, J. & Frisch, a. Exploring chemistry with electronic structure methods, 1996. *Gaussian Inc, Pittsburgh, PA* (1996).

2. Wong, M. W. Vibrational frequency prediction using density functional theory. *Chem. Phys. Lett.* **256**, 391–399 (1996).

3. Lee, C., Yang, W. & Parr, R. G. Development of the Colle-Salvetti correlation-energy formula into a functional of the electron density. *Phys. Rev. B* **37**, 785–789 (1988).

4. Patterson, J. D. Density-functional theory of atoms and molecules. *Ann. Nucl. Energy* **16**, 611 (1989).

5. Pearson, R. G. The electronic chemical potential and chemical hardness. *J. Mol. Struct. THEOCHEM* **255**, 261–270 (1992).

6. Lu, T. & Chen, F. Multiwfn: A multifunctional wavefunction analyzer. *J. Comput. Chem.* **33**, 580–592 (2012).
